# An object numbering task reveals an underestimation of complexity for typically structured scenes

**DOI:** 10.3758/s13423-024-02577-2

**Published:** 2024-09-17

**Authors:** Alex A. Carter, Daniel Kaiser

**Affiliations:** 1 Department of Psychology, University of York, York, UK; 2 Department of Mathematics and Computer Science, Physics, Geography, Justus-Liebig-Universität Gießen, Arndtstraße 2, 35392 Gießen, Germany; 3 Center for Mind, Brain and Behavior (CMBB), Philipps-Universität Marburg, Justus-Liebig-Universität Gießen, and Technische Universität Darmstadt, Hans-Meerwein-Straße 6, 35032 Marburg, Germany

**Keywords:** Scene perception, Numerosity, Complexity, Object regularities

## Abstract

Our visual environments are composed of an abundance of individual objects. The efficiency with which we can parse such rich environments is remarkable. Previous work suggests that this efficiency is partly explained by grouping mechanisms, which allow the visual system to process the objects that surround us as meaningful groups rather than individual entities. Here, we show that the grouping of objects in typically and meaningfully structured environments directly relates to a reduction of perceived complexity. In an object numerosity discrimination task, we showed participants pairs of schematic scene miniatures, in which objects were structured in typical or atypical ways and asked them to judge which scene consisted of more individual objects. Critically, participants underestimated the number of objects in typically structured compared with atypically structured scenes, suggesting that grouping based on typical object configurations reduces the perceived numerical complexity of a scene. In two control experiments, we show that this overestimation also occurs when the objects are presented on textured backgrounds, and that it is specific to upright scenes, indicating that it is not related to basic visual feature differences between typically and atypically structured scenes. Together, our results suggest that our visual surroundings appear less complex to the visual system than the number of objects in them makes us believe.

## Introduction

Vision in natural environments is highly efficient: Humans can efficiently find objects embedded in rich natural scenes (Li et al., 2002; Peelen & Kastner, 2014; Wolfe, Alvarez, et al., 2011a) and remember large amounts of information from previously viewed scenes (Konkle et al., 2010). The efficiency with which humans perform such tasks is often described as surprising or puzzling (Peelen & Kastner, 2014; Wolfe, Alvarez, et al., 2011a). This notion stems from studies with simple visual stimuli that revealed severe capacity limitations in processing multiple stimuli: Behavioral performance in such studies drops rapidly when more and more items need to be searched (Wolfe, 2010) or remembered (Luck & Vogel, 2013). Given the large number of objects contained in natural scenes, vision should be highly inefficient in most everyday situations.

A possible explanation is based on the typical structure of natural scenes (Biederman et al., 1982; Kaiser et al., 2019; Võ et al., 2019; Wolfe, Võ, et al., 2011b): In scenes, objects do not appear alone and in random locations but they form meaningful spatial arrangements. For instance, lamps appear above tables, and chairs appear next to them. To facilitate the processing of multiple simultaneous objects, the visual system may exploit the ways in which objects typically appear together in the world. Indeed, behavioral studies suggest that typical multiobject configurations facilitate visual tasks like detection (Riddoch et al., 2003; Stein et al., 2015), search (Goupil et al., 2023; Kaiser et al., 2014), or short-term memory (Kaiser et al., 2015; Liu et al., 2022; O’Donnell et al., 2018). Such effects have been explained by a grouping of objects into larger units of processing, allowing the brain to process objects at the level of (fewer) groups rather than at the level of (more) individual objects (Kaiser et al., 2019; Kaiser & Peelen, 2018).

If observers indeed process the environment on the level of meaningful object groups rather than at the level of individual objects, this raises the question of how complex the visual world really is to the visual system. The notion of rich visual environments is partly derived from estimating the number of individual objects that make up a scene, where the abundance of individual objects indexes a scene’s richness (Neider & Zelinsky, 2008; Wolfe, Alvarez, et al., 2011a). Such measures may indeed overestimate the complexity of natural scenes, as they do not take grouping processes into account (Neider & Zelinsky, 2008). Here, we sought to investigate whether typically structured environments, which enable participants to effectively group objects, are perceived as less complex than environments where typical object configurations are disrupted, hindering the grouping of objects into meaningful ensembles.

To quantify perceived complexity, we employed a numerosity discrimination paradigm. Humans are relatively accurate in estimating numerosity, even for larger quantities (Anobile et al., 2016; Feigenson et al., 2004; Kaufman et al., 1949). Recent studies show that this accuracy is preserved when objects are embedded in natural scenes (Odic & Oppenheimer, 2023; Wencheng et al., 2023). Here, we used numerosity as a proxy for visual complexity: The more objects a scene is judged to contain, the more complex it appears. This notion corresponds with judgments of scene complexity, which are most strongly driven by the number of objects or the overall clutter in a scene (Olivia et al., 2004).

**Fig. 1:**
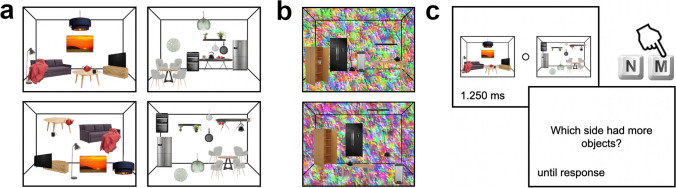
Stimuli and paradigm. **a** Stimuli were schematic scene miniatures (kitchens and living rooms), consisting of 10 to 20 individual objects. Scenes could be typically structured, resembling real-world regularities in object configurations (top row), or atypically structured, with object locations shuffled (bottom row). Examples show rooms with 10 objects (left column) or 20 objects (right column). **b** In Experiment 2, the same scene miniatures were shown on a colored texture background, as illustrated. **c** On each experimental trial, participants were asked to judge which of two simultaneously presented scenes contained more objects

In our study, participants judged which of two schematic miniature scenes had the greater number of objects in them. Critically, we presented participants with scenes in which the objects were presented in accordance with typical real-world structure and scenes where real-world structure was violated by shuffling object locations across the scene (Fig. 1). With this paradigm, we tested two critical hypotheses. First, we hypothesized that the discrimination of object numerosity in typically structured scenes should be worse than the discrimination of numerosity in atypically structured scenes, as grouping processes in typically structured scenes hinder the effective individuation of items. Second, and more critically, we hypothesized that object numerosities should be underestimated in typically structured, compared with atypically structured, scenes, as grouping processes in typically structured scenes lead to an aggregation of individual objects into fewer groups and thereby reduce the perceived object count.

## Methods

### Participants

We conducted three experiments. In Experiment 1, we tested 34 participants. Data from one participant was lost due to an error in data saving, leaving a final sample of 33 participants (nine men, 24 women; mean age = 26.9 years, *SD* = 4.2). In Experiment 2, we tested another 34 participants (eight men, 26 women; mean age = 26.1 years, *SD* = 4.4). In Experiment 3, we tested 36 participants. One participant in Experiment 3 was excluded because they did not perform the task correctly, leaving a final sample of 35 participants (12 men, 23 women; mean age = 26.7 years, *SD* = 4.4). Sample sizes were chosen based on convenience sampling, with the goal of *n* ≥ 34 to achieve a power of 80% for uncovering medium sized effects (Cohen’s *d* = 0.5) in a *t* test. All participants had normal or corrected-to-normal visual acuity. They received a monetary compensation for their participation. Each participant provided written informed consent prior to the experiments. Procedures were approved by the general ethical committee of the Justus Liebig University Gießen and in accordance with the Declaration of Helsinki.

### Stimuli

The stimulus set consisted of schematic scene miniatures from two categories (kitchens and living rooms). We constructed a typical and an atypical version for each scene. The typically structured versions were constructed by superimposing a set of isolated and colored real-world objects on top of a black perspective grid in a way that resembles the typical arrangement of the objects in a typical kitchen or living room (Fig. 1a). Each scene consisted of 10 to 20 individual objects (i.e., 11 different object numerosities). For each numerosity, we constructed two exemplars per category, yielding a total of 22 unique typically structured scenes per category (44 in total). The atypically structured versions were generated by shuffling the objects around in space (Fig. 1a). This shuffling was done manually by the authors while approximately controlling for the eccentricity and overlap of objects. The percentage of colored pixels in both the kitchens and living rooms only differed very slightly between typically and atypically structured scenes (kitchens: 19.7% versus 19.9%; living rooms: 26.7% versus 27.5%), indicating that we did not introduce substantial image-based confounds. The stimulus set in total consisted of 22 unique atypically structured scenes per category (44 in total).

### Paradigm–Experiment 1

The experiments were coded in Psychtoolbox for MATLAB (Brainard, 1997). During each experimental trial, participants viewed a pair of scenes, with one scene on each the left and right sides of the screen. The scenes were presented for 1.25 s. Each scene subtended approximately 17° × 13° visual angle, with scenes presented approximately 5° away from the center of the screen. Participants were tasked with judging which of the scenes had more objects in them by pressing the “n” or “m” keys on the keyboard corresponding to the left and right stimulus, respectively (Fig. 1c). Responses were recorded during a response prompt appearing after the scene display, and participants were asked to respond as accurately as possible. Trials were separated by a 500-ms intertrial interval.

Before the experiment, participants were instructed that the notion of an object in the context of the experiment refers to all nameable, separable objects contained in the scene. The experiment started with a practice block of five trials to familiarize people with the displays and response keys. These five trials featured different scenes than the ones used in the subsequent experiment, and they were discarded from all analyses.

In the subsequent experiment, each trial always featured two images of the same category (i.e., two kitchens or two living rooms). The other conditions were fully balanced: That is, each numerosity was once paired with each other numerosity, once for two typically structured scenes, once for two atypically structured scenes, and twice for a typically and an atypically structured scene (once with the typically structured scene on the left and once with the typically structured scene on the right). This yielded 2 (category) × 11 (numerosity left) × 11 (numerosity right) × 4 (typicality combination) trials—that is, 968 trials in total. On each trial, one exemplar of the two available exemplars from each category and at each numerosity was chosen randomly. Trials with the same numerosity never showed the exact same scene but the two different exemplars available. Trial order was fully randomized. The experiment lasted 45 min and was divided into four blocks.

### Paradigm–Experiment 2

Experiment 2 was identical to Experiment 1, apart from one critical change. Instead of presenting the scene miniatures on a white background, we superimposed them on a colored texture background (Fig. 1b). For this, we used textures that were previously used as visual masks (Kaiser et al., 2016). We used a total of 20 backgrounds and randomly selected two backgrounds for every trial so that the backgrounds on the left and right sies of the displays were never identical.

### Paradigm–Experiment 3

Experiment 3 was identical to Experiment 1, apart from the following changes: First, we only included trials that featured a typically and an atypically structured scene. Thus, there were no trials where a typical scene was compared with another typical scene, or an atypical scene was compared with another atypical scene. Second, we additionally included an equal number of trials where the scenes were presented in an upside-down orientation (i.e., inverted), to test whether the effects obtained in Experiment 1 were indeed due to typicality or rather caused by low-level feature differences between the typically and atypically structured scenes (Kaiser et al., 2014; Stein et al., 2015). This yielded 2 (category) × 11 (numerosity left) × 11 (numerosity right) × 2 (typicality combination) × 2 (orientation) trials—that is, 968 trials in total. The experiment again lasted 45 min, split into four blocks.

### Data analysis–Experiments 1 and 2

Data analysis proceeded identically for Experiments 1 and 2. Data were analyzed by fitting psychometric functions to the behavioral responses, separately for each participant. Specifically, we used the Palamedes Toolbox (Prins & Kingdom, 2018) to fit cumulative Gaussian functions to individual participants response data, using a maximum likelihood criterion. Psychometric functions were fit on two separate parts of the data (see below). For all analyses, data from the two scene categories were collapsed to yield more data for fitting the psychometric functions.

First, we focused on the trials in which two typically structured or two atypically structured scenes were presented. Here, response data were recoded to obtain responses as a function of the ratio of the number of objects in the stimulus presented on the right side of the display versus the stimulus on the left side of the display. Ratios $r$ were obtained from object numerosity $n$ using the following formula:

$$r= \left\{\begin{array}{lc}\frac{{n}_{right}}{{n}_{left}}\ \ \ \ \ \ \ \ \ \ \ \text{for}\ {n}_{right}\ \ \ \ \ \ \ \ \ \ \ge {n}_{left} \\ 2-\frac{{n}_{left}}{{n}_{right}}\ \ \ \ \text{for}\ {n}_{right}\ \ \ \ \ \ \ \ \ <{n}_{left}\end{array}\right.$$

when interpreting these values, a ratio of $r=2$ thus indexes twice as many objects on the right, whereas a ratio of $r=0$ indexes twice as many objects on the left. Ratios were rounded to the first decimal point (i.e., multiples of 0.1). We then plotted the data as a function of responses choosing the right-side stimulus as a function of the relative difference in numerosity between the left and right stimuli and fitted a cumulative Gaussian function. This was done separately for the typically and atypically structured scenes. We fitted two parameters: the slope (the variance of the Gaussian distribution) and the point of subjective equality (PSE; the mean of the Gaussian distribution) for each fit. These parameters were estimated separately for each participant. In this analysis, the slope indicates how well participants could discriminate between numerosities, while a non-zero PSE indicates a bias for overestimating numerosity on one side of the display.

Second, we focused on the trials in which a typically structured scene and an atypically structured scene were presented. Here, response data were recoded to obtain responses as a function of the ratio of the number of objects in the typically structured and the atypically structured scenes (using the same formula as above). Responses were similarity fit with a cumulative Gaussian function, yielding a slope and PSE for assessing the direct comparison between the typically and atypically structured scenes. In this analysis, the slope indicates how well participants could discriminate between numerosities, while a non-zero PSE indicates a bias for overestimating numerosity in either the typically or atypically structured scenes.

### Data analysis–Experiment 3

Experiment 3 only featured trials in which a typically structured scene and an atypically structured scene were presented. Response data were thus again recoded to obtain responses as a function of the ratio of the number of objects in the typically structured and the atypically structured scenes. Responses were similarity fit with a cumulative Gaussian function, yielding a slope and PSE for assessing the direct comparison between the typically and atypically structured scenes. As in the second analysis for Experiment 1, a non-zero PSE indicates an overestimation of numerosity for the typically or atypically structured scenes. Here, we performed this analysis separately for the upright and inverted scenes, allowing us to compare the parameters across scene orientations.

### Statistical analysis

Slope and PSE parameters were compared against zero and one, respectively, and between conditions using one- or two-sample *t* tests, respectively. All *t* tests were two sided. Cohen’s *d* is provided as a measure of effect size.

### Open practices statement

All materials, data, and code are available on the Open Science Framework (https://osf.io/hkxur/).

## Results

### Experiment 1

In Experiment 1, participants judged which of two scenes presented on the right and left sides of the screen contained more objects. Scenes on either side of the display could either be typically structured or atypically structured (with object positions shuffled across space).

**Fig. 2:**
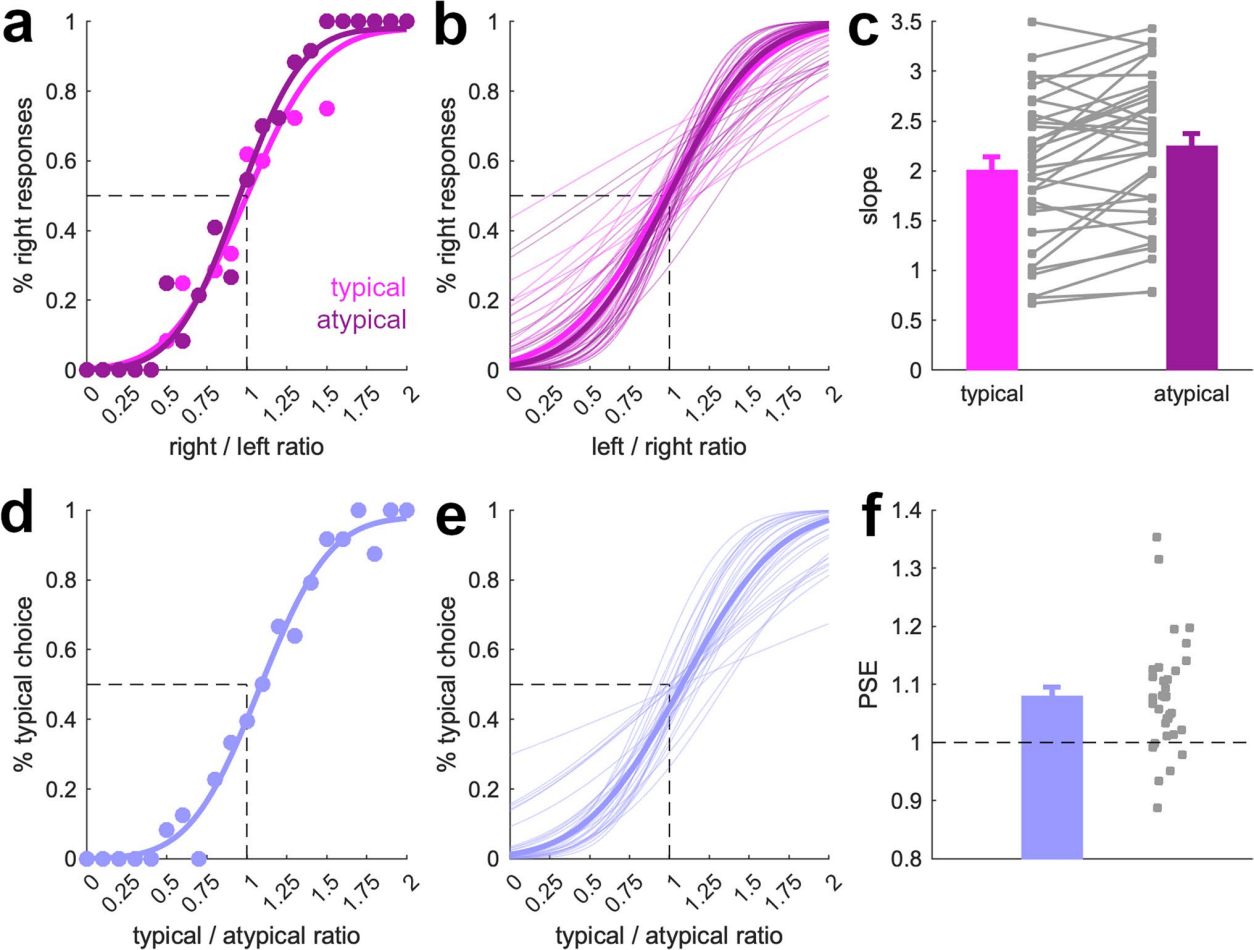
Results from Experiment 1. First, we fitted participant-specific psychometric functions to trials in which either two typically structured scenes or two atypically structured scenes were shown. **a** Data and psychometric functions for an example participant. **b** Psychometric functions based on the average slope and PSE across participants (bold lines) and for all individual participants (fine lines). **c** Slopes were shallower when object numerosities were discriminated between two typically structured scenes than when they were discriminated between two atypically structured scenes. Error bars show standard errors of the mean. Gray squares are data from individual participants. Second, we fitted psychometric functions to trials in which a typically structured scene was shown together with an atypically structured scene. **d** Data and psychometric function for an example participant. **e** Psychometric functions based on the average slope and PSE across participants (bold lines) and for all individual participants (fine lines). **f** PSEs were shifted positively, indicating an underestimation of numerosity in typically structured, compared with atypically structured, scenes. Error bars show standard errors of the mean. Gray squares are data from individual participants

First, we analyzed data from trials where participants compared two typically structured scenes with each other or two atypically structured scenes with each other. In both conditions, participants were able to tell apart the numerosities of the two scenes: When fitting psychometric functions (see Methods), all participants produced positive slopes: mean slope for typically structured scenes = 2.0, *SD* = 0.75), comparison against 0, *t*(32) = 15.3, *p* < .001, *d* = 2.7; mean slope for atypically structured scenes = 2.2, *SD* = 0.75; comparison against 0, *t*(32) = 17.3, *p* < .001, *d* = 3.0, and there was no overall bias towards overestimating numerosities on either side of the display: mean PSE for typically structured scenes = 0.97, *SD* = 0.16; comparison against 1, *t*(32) = 0.9, *p* = .38, *d* = 0.15; mean PSE for atypically structured scenes = 0.99, *SD* = 0.14; comparison against 1, *t*(32) = 0.2, *p* = 0.83, *d* = 0.04. We hypothesized that the grouping of individual objects into meaningful arrangements leads to a less accurate individuation of objects and thus diminished sensitivity for trials featuring typically structured, compared with atypically structured, scenes. This hypothesis was confirmed by a significantly shallower slope of the psychometric function for trials featuring typical scenes than trials featuring atypically structured scenes, *t*(32) = 3.6, *p* = .001, *d* = 0.63 (Fig. 2a–c). PSEs did not differ between conditions, *t*(32) = 1.0, *p* = .33, *d* = 0.17.

Second, we analyzed data from trials where participants compared a typically structured scene to an atypically structured scene, independently of whether the typically structured scene appeared on the right or left side of the screen. Here, we hypothesized that participants would underestimate the numerosity of typically structured scenes compared with atypically structured scenes, as a grouping of individual objects would hinder object individuation in the typically structured scenes. This hypothesis was confirmed by a significantly shifted PSE in the psychometric function, *t*(32) = 4.7, *p* < .001, *d* = 0.82 (comparison of the PSE against 1; Fig. 2d–f).

The results of Experiment 1 show that the number of objects contained in typically structured scenes is less accurately estimated and that the number of objects contained in typically structured scenes is underestimated relative to atypically structured scenes. These findings suggest that grouping processes specific to typically structured scenes reduce their perceived complexity.

### Experiment 2

Although we paid close attention to not introducing changes in visual features like the eccentricity or overlap of objects, we could still have introduced low-level differences that can explain the underestimation of numerosity in the typical scenes. One such difference could be that a different number of nonwhite pixels across the white background may provide a powerful cue for solving the task. To assess whether such a “shortcut” towards estimating numerosity explains the results from Experiment 1, we showed the scene miniatures on top of a colored texture background instead of a plain white background. Otherwise, the experiment was identical to Experiment 1.

**Fig. 3:**
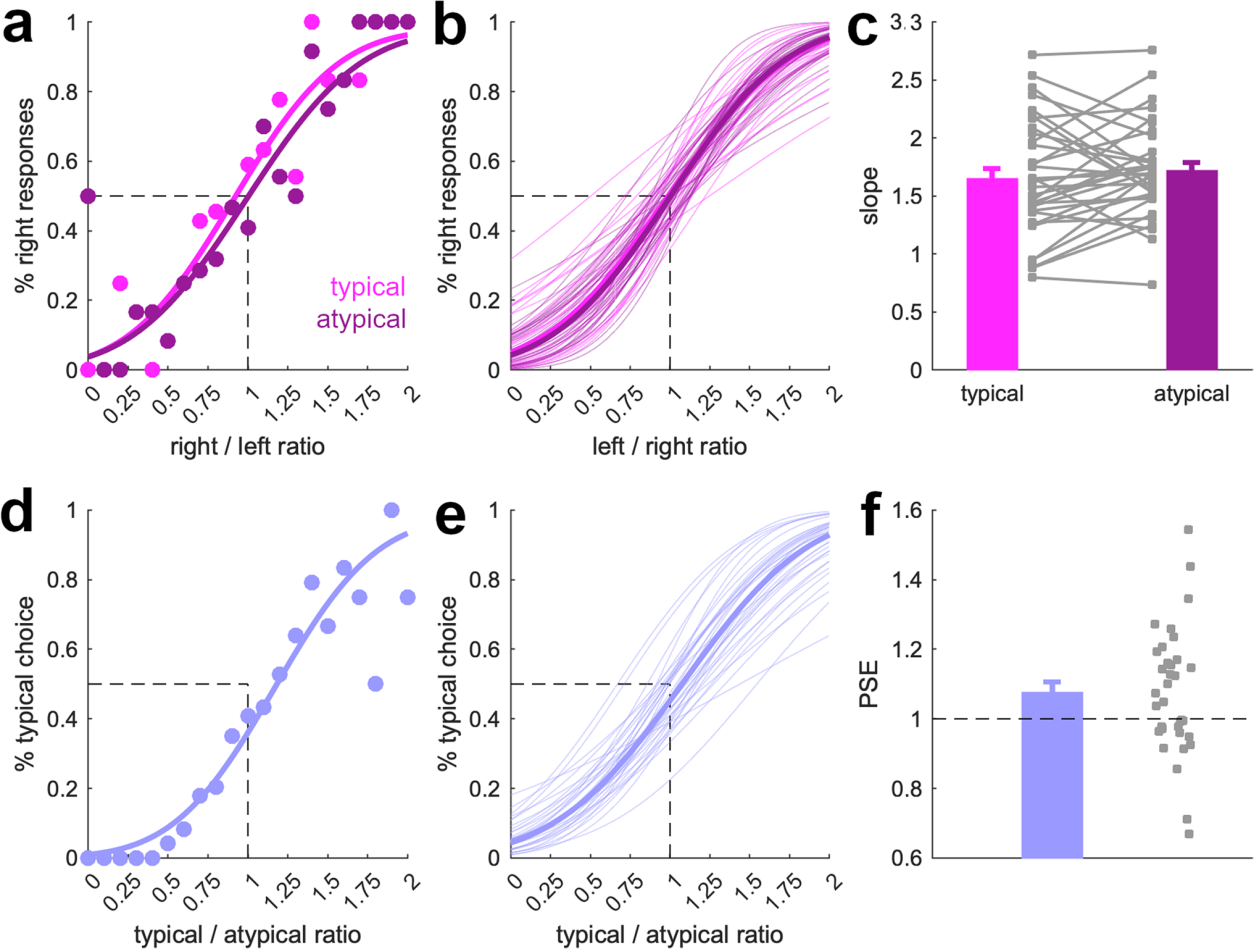
Results from Experiment 2. First, we fitted participant-specific psychometric functions to trials in which either two typically structured scenes or two atypically structured scenes were shown. **a** Data and psychometric functions for an example participant. **b** Psychometric functions based on the average slope and PSE across participants (bold lines) and for all individual participants (fine lines). **c** Slopes were shallower when object numerosities were discriminated between two typically structured scenes than when they were discriminated between two atypically structured scenes. Error bars show standard errors of the mean. Gray squares are data from individual participants. Second, we fitted psychometric functions to trials in which a typically structured scene was shown together with an atypically structured scene. **d** Data and psychometric function for an example participant. **e** Psychometric functions based on the average slope and PSE across participants (bold lines) and for all individual participants (fine lines). **f** PSEs were shifted positively, indicating an underestimation of numerosity in typically structured, compared with atypically structured, scenes. Error bars show standard errors of the mean. Gray squares are data from individual participants

Participants could successfully perform the task despite the textured background, as indicated by positive slopes in all conditions, all *t*(33) > 19.1, *p* < .001, *d* > 3.2. As expected, slopes were, however, shallower than in Experiment 1, all *t*(33) > 2.29, *p* < 0.03 (independent-samples *t* tests). When looking at the difference in slopes between the trials where two typically structured and atypically structured scenes were compared, we could not replicate the effect obtained in Experiment 1 (Fig. 3a–c): Slopes were not significantly different between the two conditions, *t*(33) = 0.95, *p* = .35, *d* = 0.16. The PSEs across conditions were not different either, *t*(33) = 0.61, *p =* .54, *d* = 0.10. This does not confirm our hypothesis of a more imprecise numerosity representation for typical scenes and indicates that the effect is not replicable or much smaller in size than in Experiment 1.

Critically, when assessing the trials where a typically and an atypically structured scene were compared, we replicated the pattern from Experiment 1 (Fig. 3d–f): Numerosity in typically structured scenes was underestimated compared with atypically structured scenes, indicated by a shift in the PSE, *t*(33) = 2.4, *p* = .022, *d* = 0.41.

### Experiment 3

In Experiment 3, we aimed to provide another, more thorough test for low-level visual confounds. We replicated Experiment 1 but only featuring trials in which a typically structured scene was paired with an atypically structured scene. Critically, each trial was once presented with upright scenes and once with inverted scenes. If the differences between typically and atypically structured scenes would indeed stem from low-level feature differences, they should be preserved across orientations. If the differences are specific to the upright conditions, then they are related to the unique possibility of grouping objects in the upright and typically structured scenes (Kaiser et al., 2014; Stein et al., 2015).

**Fig. 4:**
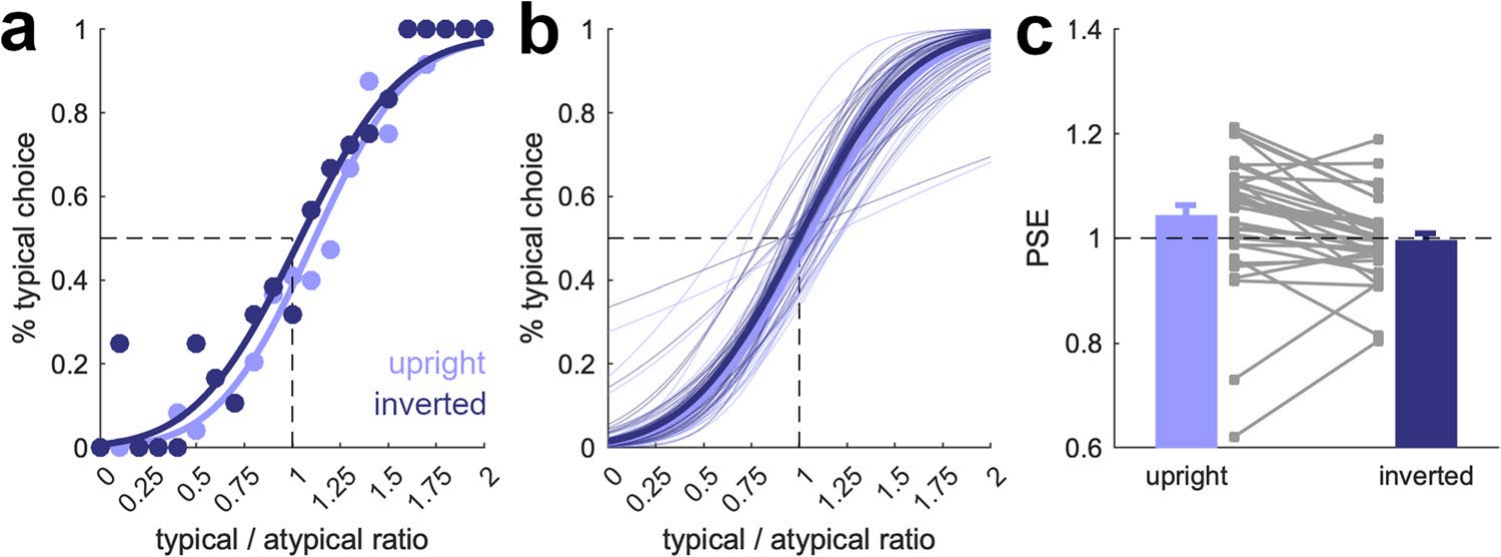
Results from Experiment 3. Here, we examined trials, in which a typically structured scene was shown together with an atypically structured scene (as in Fig. 2d–f). We fitted participant-specific psychometric functions separately for trials in which the scenes were upright or inverted. **a** Data and psychometric functions for an example participant. **b** Psychometric functions based on the average slope and PSE across participants (bold lines) and for all individual participants (fine lines). **c** For the upright scenes, PSEs were shifted positively, replicating the result from Experiment 1. This shift was absent for inverted scenes. Error bars show standard errors of the mean. Gray squares are data from individual participants

Here, we fitted psychometric functions separately for trials with upright and inverted scenes. For the upright scenes, we again found a significantly shifted PSE, *t*(34) = 2.1, *p* = .045, *d* = 0.35 (comparison of the PSE against1; Fig. 4), replicating the results from Experiment 1 and indicating that the number of objects in typically structured scenes was underestimated compared with atypically structured scenes. The effect was less pronounced than in Experiment 1, but this difference can be attributed to two outlier participants with strong negative shifts in their PSE (see Fig. 3c). Critically, we did not find a similar shift in the PSE for inverted scenes, *t*(34) = 0.3, *p* = .74, *d* = 0.06, and the shift in the PSE was significantly greater for the upright than for the inverted scenes, *t*(34) = 3.2, *p* = .002, *d =* 0.55. Slopes did not differ between conditions, *t*(34) = 0.5, *p* = .62, *d* = 0.09. This suggests that object individuation is not necessarily impacted by inverting a scene.

The results from Experiment 3 replicate the underestimation of object numerosity in typically structured scenes observed in Experiments 1 and 2. They further show that the reduction in perceived complexity for typically structured scenes cannot be explained by low-level differences between the typically and atypically structured scenes in our stimulus set.

## Discussion

Our study used an object numerosity discrimination paradigm to quantify the perceived complexity of naturalistic scenes (schematic miniatures of living rooms and kitchens). We specifically tested whether typically structured scenes are perceived as less numerically complex—that is, whether the number of objects is underestimated in typically structured, compared with atypically structured scenes. Across three experiments, when comparing numerosity between a typically and atypically structured scene, the typically structured scene needed more objects to be judged as containing an equal number of objects to an atypically structured scene. This shows that grouping processes lead to a relative underestimation of object numerosity when scenes are structured in line with real-world regularities and thus allow for effective grouping of objects. This effect was replicated when the objects were shown on a textured background (rather than a uniformly white background) and was abolished when the scenes were viewed upside down. This shows that the effect is not related to low-level visual differences between the typically and atypically structured scenes. Together, these results show that participants reliably underestimate the complexity of a scene’s object content when a scene is typically structured, suggesting that grouping processes render scenes less complex to the visual system than they appear to be.

We also hypothesized that participants should be less sensitive to object numerosity when comparing two typically structured scenes, relative to comparing two atypically structured scenes, because object grouping processes should to some extent hamper the correct individuation of objects. While such an effect was observed in Experiment 1, it was not replicated in Experiment 2. From our data, we thus cannot draw any firm conclusions about whether object numerosity estimation is generally worse in typically structured compared with atypically structured scenes. While the true effect may simply be much smaller than the relatively big effect obtained in Experiment 1, the difference in results may also be related to the presence of a textured background in Experiment 2. If, and under which conditions, numerosity judgments differ between typically and atypically structured scenes are reliably observed needs to be clarified in future work.

Our findings support previous observations that grouping processes are specifically observed when objects adhere to real-world regularities (Kaiser et al., 2019). Here, we provide a novel measure for testing how such grouping processes impinge on the perceived complexity of scenes: When objects contained in typically structured scenes can be grouped, they are judged as containing fewer objects than when scenes are atypically structured. This finding is compatible with reports of an underestimation of numerosity when simple stimuli can be grouped on the basis of Gestalt laws (Chakravarthi et al., 2023; Franconeri et al., 2009; He et al., 2009, 2015; Im et al., 2016). However, our results are not readily explained by such low-level grouping processes: When scenes were inverted, the underestimation of numerosity in typically structured scenes was not observed, indicating that low-level grouping processes, which should operate across stimulus orientations, did not drive the effect. It would nonetheless be interesting to explore if simplified stimuli that mimic the distribution of information across space (such as meaningless silhouettes that are spatially arranged to mimic real scenes) can produce similar effects as the ones reported here.

Our findings further have implications for quantifying the complexity of natural scenes by simply counting the individual objects in a scene. It has been argued that such approaches, if anything, underestimate the number of objects contained in a scene, as objects can often be broken down into meaningful parts, which would render the number of objects even higher (Wolfe, Alvarez, et al., 2011a). Our findings suggest the contrary: Counting individual objects may overestimate the complexity of natural scenes, as grouping processes change the units of processing from (more) individual objects to (fewer) groups of objects. Future studies could test whether taking such grouping processes into account when estimating a scene’s complexity can better align models of visual processing from simple visual stimuli with data from more naturalistic experiments.

Another relevant issue that needs to be addressed by future work is which types of object statistics are most critical for the reduction in complexity. Candidate statistics include the absolute spatial position of individual objects (Biederman et al., 1982; Kaiser & Cichy, 2018), the grouping of multiple objects into meaningful arrangements (Kaiser et al., 2014; Stein et al., 2015) and the adherence of object positioning to the laws of physics (Biederman et al., 1982; Võ & Henderson, 2009). The current study aimed for a strong manipulation that conflates these different factors.

It is worth noting that the underestimation of object numerosity in our study was numerically not dramatic. However, our task forced participants to individuate separate objects. This individuation may override grouping processes to some extent, leading to an underestimation of grouping effects in scenarios where individuation is not explicitly required. In many real-world situations, groups of objects are entirely task irrelevant, so that they can be easily suppressed to facilitate the processing of task-relevant information (see Kaiser et al., 2014). In such situations, complexity may be reduced much more drastically than in our task.

Finally, our study uses a very specific class of stimuli: schematic scene miniatures that are mimicking the structure of natural scenes but are not faithfully resembling all aspects of everyday environments. Future studies need to test whether similar effects can be found in real-world scene photographs that are structured to varying degrees. Furthermore, it will be interesting to see whether our results extend to grouping processes for other visual content, such as action-related grouping of objects (Humphreys & Riddoch, 2007) or social relations among human agents (Papeo, 2020).

Together, our study provides novel evidence for an underestimation of object numerosity in structured natural scenes. This underestimation likely mirrors a reduction of the effective complexity of natural scenes, caused by object grouping processes in the visual system. This reduction of complexity may be critical for adaptive visual cognition in real-world environments.

## Data Availability

All materials, data, and code are available on the Open Science Framework (https://osf.io/hkxur/). The experiments were not preregistered.
